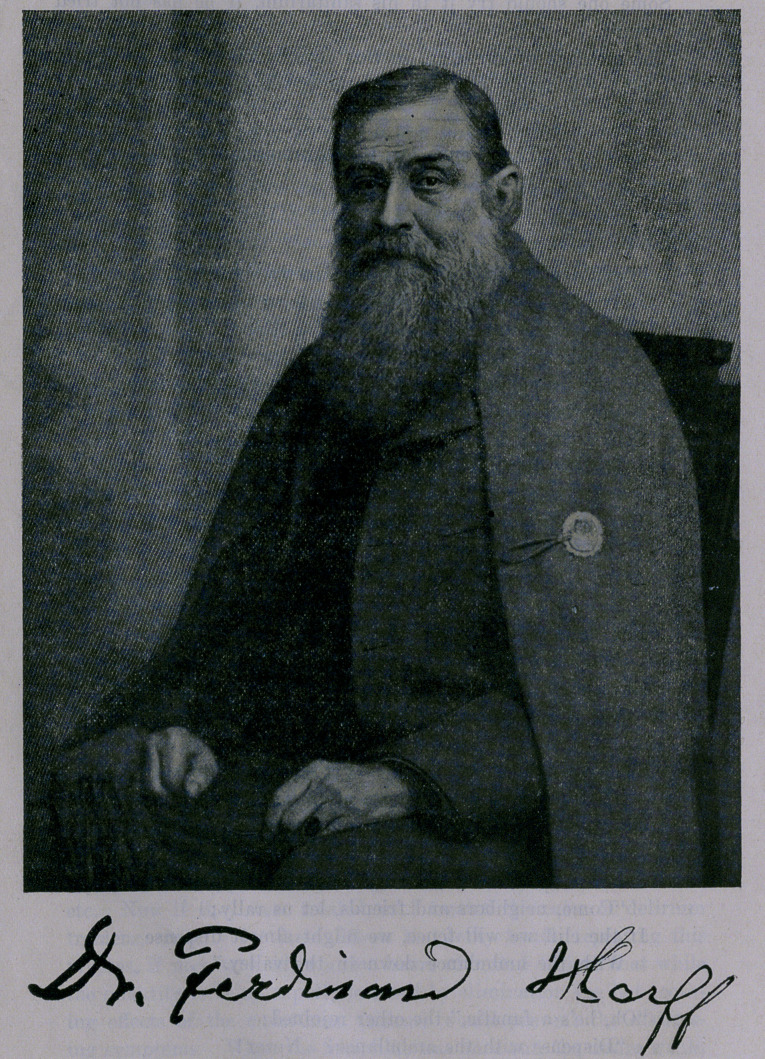# Death of Doctor Herff

**Published:** 1912-06

**Authors:** R. H. L. Bibb



Dr. Ferdinand Herff

### EDITORIAL DEPARTMENT

**DR. F. E. DANIEL, Editor** **DR. R. H. L. BIBB, Associate Editor**

This issue of the "Red Back" completes its twenty-seventh year, under one—the original—management. It makes its bow to its hosts of friends and loyal supporters—the best element of the Texas profession, and the best of the advertising patronage—much of which has been with me more than a quarter century—thanks them and cranks up for the twenty-eighth-mile run—full of hope and enthusiasm for the betterment of that profession to which its founder has given fifty years of a very busy life.

---

### DEATH OF DOCTOR HERFF.

The death of Dr. Ferdinand Herff, at the advanced age of 92 years, at his home in San Antonio, Texas, May 18, 1912, marks the passing away of one of the most noted, beloved, dexterous and successful surgeons that ever adorned the annals of American surgery in the South.

Dr. Herff was born in the city of Hesse, Darmstadt, November 29, 1820. His father, a nobleman, was judge of the Supreme Court of Hesse, Darmstadt. His family was of Belgian origin, but fled from that country in 1568, to escape the persecutions of the savage and blood-thirsty Duke of Alva.

After completing his studies in the Darmstadt Gymnasium (College), young Herff spent two years at the University of Bonn, of which an uncle, a diplomat and author of distinction, was president. In his uncle's family he met many of the nobles and notables of that day, among whom was the illustrious Alexander von Humboldt, Prince Albert, the Duke of Coburg, consort of Queen Victoria, and Prince Frederick of Prussia, afterwards Emperor of Germany. The two following years he passed at the University of Berlin, but was forced, by the then existing laws, to spend his last two years and to pass his final examination, which he did, at Giessen, his home university, from which he graduated in 1843. Upon graduation he was tendered the chair of surgery in his Alma Mater, which he declined, in order to accept the position of surgeon in the Hessian army, where he soon became famous for his brilliant work in plastic surgery and in the surgical treatment of tubercular abscesses of the lungs, which fame followed him for years and was signally recognized by the eminent Virchow, who met him years afterwards and expressed delight in meeting Dr. Herff, of whom he had heard so many flattering accounts.

Becoming imbued with the communistic ideas which were animating the liberty-loving throughout Europe about this time, Dr. Herff and other university friends organized a movement to establish a communistic colony in America. To this end he obtained leave of absence from the army, and with numerous young university graduates, noblemen and others, set out for Texas, where they arrived and settled on the Llano river, near Castell, in 1847. Here instead of pursuing his studies in botany and the other sciences, as he intended, the doctor literally put his hands to the plow and did all kinds of manual labor until he finally decided to make Texas his future home. With this determination he went back to his native land, in 1848, to marry his betrothed, the accomplished Miss Kingelhoffer, of Giessen, with whom he returned as his beautiful and loving bride, in 1849, and settled in New Braunfels, where he remained until April, 1850, when he located permanently in San Antonio—a little village then of some 3000 inhabitants—where he began one of the largest, most remarkable and successful careers in the history of medicine.

When Dr. Herff reached San Antonio he had only 10 cents in money; and he had hardly alighted from the wagon which brought him before he was importuned for alms by a little Mexican girl, who was leading an old blind beggar, half-starved and clothed in rags and tatters, to whom, with that nobleness of soul and utter forgetfulness of self whenever and wherever he could minister to the ills and sufferings of others, which made the doctor's life so beautiful and so charming, he gave the dime, the last cent he had, telling her that he had intended to buy himself a steak for supper with it, but that the old man looked as if he needed it most. When Dr. Herff cast this morsel of bread upon the waters, he little reckoned that ere the rising of another sun it would be returned to him fifty-fold, but it was, for during the night he was called to a man in great suffering, whom he quickly relieved, and was rewarded with a $5.00 fee by his grateful patient.

In 1866, in company with his family, Dr. Herff visited his fatherland again and for the last time, and, as Austria and Prussia were at war, he enjoyed exceptionally good opportunities, of which he availed himself, to engage in military hospital practice. He returned to San Antonio in 1867, and began the third, and possibly the most fruitful period of his medical career. His fame as a physician and surgeon spread far and near. He performed all of the major operations in surgery with a dexterity and success surpassed by no one; whilst his skill as a physician was recognized everywhere.

In early days, Dr. Herff was a contract surgeon in the United States army; he was a brigade surgeon in the Confederate militia during the Civil War; city physician of San Antonio in 1855—upon the munificent salary of $10 a month; member of the Hessian Association of Physicians and Surgeons, and of the Darmstadt Society of Natural Sciences. He assisted in organizing the Texas Medical Association in 1853. He was a charter member of the West Texas Medical Association, which was organized in 1876, and was afterwards, in 1892, elected a life member of that body. Upon reorganization of the Bexar County Medical Society he was elected an honorary member. The St. Louis College of Physicians and Surgeons conferred its honorary degree of Doctor of Medicine upon him in 1882. He was tendered the professorship in surgery in the Medical Department of the Texas State University in 1891, but whilst deeply sensible of the great honor he could not be prevailed upon to accept it. His Alma Mater, the University of Giessen, presented him with its honorary degree in 1893 in commemoration of the fiftieth anniversary of his graduation from that institution. This was a tribute of which the doctor was justly very proud. He discovered the hookworm in Texas in 1864. Being familiar with that parasite in Europe, and an adept in microscopy, he had no difficulty in recognizing the first case of it which he saw in this country.

On May 1, 1905, there was unveiled in the Carnegie Public Library, and presented to the city of San Antonio, a beautiful bronze bust of Dr. Herff. This loving tribute came from contributions from hundreds of the doctor's grateful patients.

Dr. Herff was as innocent and as guileless as a child, as modest as a little girl, and as tender as a mother, and yet, withal, he was as firm as the ages and as brave as the bravest. Wherever there was a human tear to dry, a pain to ease or a troubled heart to soothe, it was there that the nobleness of his character shone most resplendent. He was a model man, a loving, indulgent husband and father, a steadfast friend, an exemplary citizen, a dexterous surgeon, a skillful physician and ever the benefactor of the poor and needy. He filled, to completeness, the mould of all that is good and noble and true in man, and he passed away—for men of

Dr. Herff's type never die—as he had lived, loved, honored and respected by all who knew him.

Five worthy sons and a multitude of loving friends mourn his going.

Peace to thy sacred ashes, my noble old friend; eternal rest to thy immortal soul.

R. H. L. Bibb.

## Figures and Tables

**Figure f1:**